# Evidence for Involvement of ADP-Ribosylation Factor 6 in Intracellular Trafficking and Release of Murine Leukemia Virus Gag

**DOI:** 10.3390/cells13030270

**Published:** 2024-01-31

**Authors:** Hyokyun Kang, Taekwon Kang, Lauryn Jackson, Amaiya Murphy, Takayuki Nitta

**Affiliations:** 1 Department of Biology, Savannah State University, Savannah, GA 31404, USA; hkang36906@med.lecom.edu (H.K.); tkang@student.savannahstate.edu (T.K.); ljacks63@student.savannahstate.edu (L.J.); amurph16@student.savannahstate.edu (A.M.); 2 Department of Molecular Biology and Biochemistry, Cancer Research Institute, University of California, Irvine, CA 92697, USA

**Keywords:** murine leukemia virus, ADP-ribosylation factor 6, PI(4,5)P_2_, phospholipase D, phosphatidylinositol-4-phosphate 5-kinase, autophagy, phosphoinositide 3-kinases, mammalian target of rapamycin, vacuolar-type ATPase, proteasome inhibitors

## Abstract

Murine leukemia viruses (MuLVs) are simple retroviruses that cause several diseases in mice. Retroviruses encode three basic genes: *gag*, *pol*, and *env.* Gag is translated as a polyprotein and moves to assembly sites where viral particles are shaped by cleavage of poly-Gag. Viral release depends on the intracellular trafficking of viral proteins, which is determined by both viral and cellular factors. ADP-ribosylation factor 6 (Arf6) is a small GTPase that regulates vesicular trafficking and recycling of different types of cargo in cells. Arf6 also activates phospholipase D (PLD) and phosphatidylinositol-4-phosphate 5-kinase (PIP5K) and produces phosphatidylinositol-4,5-bisphosphate (PI(4,5)P_2_). We investigated how Arf6 affected MuLV release with a constitutively active form of Arf6, Arf6Q67L. Expression of Arf6Q67L impaired Gag release by accumulating Gag at PI(4,5)P_2_-enriched compartments in the cytoplasm. Treatment of the inhibitors for PLD and PIP5K impaired or recovered MuLV Gag release in the cells expressing GFP (control) and Arf6Q67L, implying that regulation of PI(4,5)P_2_ through PLD and PIP5K affected MuLV release. Interference with the phosphoinositide 3-kinases, mammalian target of rapamycin (mTOR) pathway, and vacuolar-type ATPase activities showed further impairment of Gag release from the cells expressing Arf6Q67L. In contrast, mTOR inhibition increased Gag release in the control cells. The proteasome inhibitors reduced viral release in the cells regardless of Arf6Q67L expression. These data outline the differences in MuLV release under the controlled and overactivated Arf6 conditions and provide new insight into pathways for MuLV release.

## 1. Introduction

Murine leukemia viruses (MuLVs) are simple retroviruses that cause several diseases, including T lymphoma, erythroid/myeloid leukemias, and spongiform encephalopathy in mice. This prototypical gammaretrovirus encodes three basic genes: *gag*, *pol*, and *env*. Gag is the structural protein of retroviral particles; Pol comprises protease, reverse transcriptase, and integrase; and Env produces membrane proteins (gp70 and p15E in MuLV) [1]. The replication cycle of MuLV is initiated by the binding of gp70 surface glycoprotein to receptors on the host cell surface. This binding causes changes in Env and induces conformational rearrangement of the p15E transmembrane protein. The viral membrane fuses with the plasma membrane and leads to the deposition of the contents of the virion in the cytoplasm. The viral RNA is reverse transcribed; then, viral DNA is transported to the nucleus and integrated into the host chromosomes as proviruses. In the late phase of a viral life cycle, viral RNA undergoes transcription and translation by the host cell machinery, and viral proteins move toward the plasma membrane where they assemble into progeny virus particles. Both viral and cellular factors mediate the intracellular trafficking of viral proteins toward assembly sites (e.g., L-domain, ESCRT machinery) [2]. However, the underlying mechanisms are not fully understood. The MuLV Gag protein is translated as poly-Gag/Pr65 and is proteolytically processed into four structural proteins—Matrix/p15, p12, Capsid/p30, and Nucleocapsid/p10—by the viral protease [1]. While Pr65 is sufficient to form virus-like particles, cleavage of Pr65 and conformational changes during budding and release are essential for producing virions [1]. 

ADP-ribosylation factor (Arf) is part of the Ras superfamily, a group consisting of over 150 small GTPases and low-molecular-weight guanine-nucleotide-binding proteins. The human Arf family contains Arf-like proteins, Sar proteins, and six Arf proteins, which are further categorized into three classes based on their genetic sequence [3]: class I (Arf1–3), class II (Arf4–5), and class III (Arf6). Arf1 and Arf6 show similarities in structure and are well characterized among the family because of their multifunction in cells [3]. Arf1 is involved in the forming and dissociating of COPI vehicles at/from the Golgi, recruitment of adaptor molecules to the trans-Golgi network, and export from recycling endosomes to the plasma membrane [4]. Arf6 localizes to the plasma membrane, endosomal compartments, and cytosol, which plays critical roles in both clathrin-dependent and clathrin-independent endocytosis, stimulation of actin polymerization, endosomal recycling, and phagocytosis [3,5]. Arf6 regulates intracellular trafficking by enhancing phosphatidylinositol-4,5-bisphosphate (PI(4,5)P_2_) production through the activation of phosphatidylinositol 4-phosphate 5-kinase (PIP5K) and phospholipase D (PLD) [3]. PIP5K generates PI(4,5)P_2_ by phosphorylating the 5 positions on the inositol ring of phosphatidylinositol 4-phosphate [6]. PLD1 and PLD2 catalyze the hydrolysis of phosphatidylcholine to generate phosphatidic acid and choline [5,7,8,9]. An increase in phosphatidic acid concentration in the cells stimulates the activity of PIP5K and provokes a secondary enhancement of PI(4,5)P_2_ production [10]. PI(4,5)P_2_ is a substrate for phosphoinositide 3-kinases (PI3K) and phospholipases to generate lipid signaling, including phosphatidylinositol (3,4,5)-trisphosphate, which could regulate early steps in autophagy [11].

Arf6 is associated with membranes by being GTP-dependent, and hydrolysis of GTP leads to the dissociation of Arf6 from the plasma membrane [3]. Arf6 function is regulated by GTPase-activating proteins (GAPs) and guanine nucleotide exchange factors (GEFs) like other GTPases. There are some types of methods and tools available to study Arf6, which include silencing or gene disruption of Arf6, expression of Arf6 GAPs or GEFs, Arf6 GEF inhibitors, and expression of Arf6 mutants with altered GDP/GTP binding with constitutively active or dominant negative forms. Among the tools, a constitutively active mutant of Arf6 (Arf6Q67L) that lacks GTPase activity has been widely used to analyze functions of Arf6 and impacts of PI(4,5)P_2_ on various biological activities. Arf6Q67L depletes PI(4,5)P_2_ from the plasma membrane and induces accumulation of PI(4,5)P_2_ in aberrant peripheral vesicles [3,12]. Here, we investigated how Arf6 controlled intracellular traffic and release of MuLV Gag with Arf6Q67L and some chemical inhibitors for PLD, PIP5K, proteasome, and autophagy. Our data demonstrated that Arf6Q67L suppressed MuLV release by accumulating Gag at PI(4,5)P_2_-enriched intracellular vesicles and affected viral release pathways regulated by PI3K, mammalian target of rapamycin (mTOR), and vacuolar-type ATPase (V-ATPase). 

## 2. Materials and Methods

### 2.1. Antibodies and Chemicals

Rabbit polyclonal anti-MuLV p30 antiserum was described previously [13]. Mouse monoclonal anti-HIV-1 p24^CA^ antibody (YDHIVgp24) was purchased from MyBioSource (San Diego, CA, USA). β-Tubulin was used for the loading control in western blots and was detected by rabbit anti-β-Tubulin antibody (Cell Signaling Technology, Danvers, MA, USA). For western blots, we used an anti-mouse IgG antibody conjugated with horseradish peroxidase (Thermo Fisher Scientific, Waltham, MA, USA) and an anti-rabbit IgG antibody conjugated with horseradish peroxidase (Thermo Fisher Scientific, Waltham, MA, USA). The antibodies for LC3 (Cell Signaling Technology) and p62/SQSTM1 (Proteintech Group, Inc., Rosemont, IL, USA) were used to check autophagic flux in the cells expressing Arf6Q67L. To examine the role of Arf6 in MuLV release, the following chemical inhibitors were used: UNC3230 (MedChemExpress, Monmouth Junction, NJ, USA), ML299 (Aobious Inc., Gloucester, MA, USA), FIPI (Tocris, Minneapolis, MN, USA), LY294002, wortmannin, rapamycin, and bafilomycin A1 (Cayman Chemical, Ann Arbor, MI, USA). MG132 and lactacystin were purchased from AdipoGen Life Sciences (San Diego, CA, USA). 

### 2.2. Cell Culture

The human embryonic kidney 293T cells and the mouse fibroblast NIH3T3 cells were cultured with DMEM media containing 10% fetal bovine serum, 100 IU/mL of penicillin, and 100ug/mL of streptomycin (Corning, NY, USA) at 37 °C with 5% CO_2_. 

### 2.3. DNA Constructs

To express viral Gag proteins, a MuLV Gag/Pol expression vector AKAQ188 [14] and an HIV-1-based packaging vector pCMV-dR8.74 were used. The following plasmids were obtained from Addgene (Watertown, MA, USA): pArf6-CFP (#11382) [15], pArf6(Q67L)-CFP (#11387) [15], pcDNA3 HA-Arf6 (#10834) [16], pcDNA3 HA-Arf6 ActQ67L (#10835) [16], 2PH-PLCdelta-GFP (#35142) and CD63-pEGFP C2 (#62964). The plasmids pcDNA3 (Invitrogen, Carlsbad, CA, USA) and pEGFP-N1 (TaKaRa Bio, San Jose, CA, USA) were used as transfection controls and were added to equalize the total amounts of DNA transfected.

### 2.4. Indirect Immunofluorescence Microscopy

The NIH3T3 cells were plated on glass coverslips coated with poly-L-lysin 16–20 h before transfection. The cells were transfected with AKAQ188, HA-Arf6 ActQ67L, pcDNA3, 2PH-PLCdelta-GFP, and CD63-pEGFP C2 by EcoTransfect (OZ Biosciences INC, San Diego, CA, USA) or jetPRIME (Polyplus, New York, NY, USA) and then incubated for 36 to 48 h. The cells were fixed with 4% paraformaldehyde for 20 min at room temperature. After the cells were washed with phosphate buffer saline (PBS) and blocked with PBS containing 10% calf serum, the serum against MuLV p30 was added. The cells were washed with PBS containing 1% Triton X-100, stained with the anti-rabbit IgG antibody conjugated with Alexa Fluor™ 555 fluoresce (Thermo Fisher Scientific, Waltham, MA, USA), followed by mounting with a Vectashield mounting medium (Vector Laboratories, Newark, CA, USA). The images were analyzed with an LSM800 microscope system (Carl Zeiss, San Diego, CA, USA).

To assess the colocalization of Gag and CD63, the individual cells were classified into four phenotypes with the following procedures. (1) Expression of both CD63 and Gag signals in the cells was examined. (2) Localization of CD63 was determined. The cells showing clear CD63 signals on the plasma membranes were classified as *PM*. The cells classified into the *PM* phenotype could exhibit CD63 signals in the cytoplasm. (3) The cells showing accumulated CD63 at the perinuclear region were classified as *PN*. The cells having both *PM* and PN phenotypes were not observed. (4) The cells showing the majority of the CD63 signals (dispersed or diffused) in the cytoplasm were classified as *Cyto*. (5) After localization of CD63 was determined, overwrapping of CD63 (green) and Gag (red) in the cells was determined. To estimate the colocalization of the signals, the colocalization tool in the Zeiss ZEN Blue software version 2.3 was used. The regions of interest (individual cells expressing green and red signals) were selected by hand-drawing over the images. The automated Costes method was applied to eliminate user bias in the estimation of thresholds, and Pearson’s correlation coefficient and Manders’ colocalization coefficients were obtained [17]. Colocalization of the signals was determined by Manders’ colocalization coefficients based on the expression of colocalizing objects in two quantities as opposed to only one defined by Pearson’s correlation coefficient. In addition, the analysis did not depend on a linear relationship between the signal levels of the two probes and was less sensitive regarding defining the regions of interest [17]. The cells showing scarce colocalization of the signals were classified as *NC* regardless of CD63 localization. Colocalization of Gag and PI(4,5)P_2_ was assessed with the PH domain of phospholipase C1 (PLC1) that binds to PI(4,5)P_2_ with high affinity. The individual cells showing clear PI(4,5)P_2_ signals on the plasma membranes were classified as *PM.* The other cells showing the majority of the signals in the cytoplasm were classified as *Cyto*. The cells classified into *Cyto* showed the dispersed and/or concentrated PI(4,5)P_2_ in the cytoplasm. Gag was colocalized with PI(4,5)P_2_ well on the plasma membrane or in the cytoplasm. 

The data were obtained from the two independent experiments and two or three individuals analyzed the cellular phenotypes in each trial. To reduce possible bias among the persons who classified the cells, the team shared the procedures to determine the cell phenotypes and the representative pictures of each phenotype. When the cells showed an intermediate phenotype between the two categories, two individuals discussed the localization of the signals to determine the phenotype. The members analyzed a similar number of cells, and the results were combined in each trial. One hundred or more cells were counted per trial to analyze colocalization of CD63 and Gag and seventy-five or more cells were counted per trial to analyze colocalization of PI(4,5)P_2_ and Gag. 

### 2.5. Virus Release Efficiency (%Gag Release)

To assess Gag release, 293T cells were transfected with AKAQ188 or pCMV-dR8.74 with pArf6-CFP, pArf6Q67L-CFP, or pEGFPN1. The media were replaced at 24 h post-transfection and the cells were treated with the inhibitors. Both the cells and media were harvested after an additional 24 h of incubation. Co-treatment with MG132 and lactacystin involved media replacement at 48 h post-transfection, followed by an additional 8 h of incubation. Equal volumes of cellular and viral samples were loaded into the acrylamide gels. Detection of intracellular Gag and released viruses using anti-p30 antibodies was described previously [18]. To quantify viral release efficiency (%Gag release), each Gag band in the cells and media was quantified with the densitometry software AlphaEaseFC version 3.1 (Alpha Innotech, San Leandro, CA, USA) and the Image J version 1.54g. The sum of all Gag species in the media divided by the sum of all Gag species in both the media and the cell lysate was used to calculate %Gag release. Different exposures of the blots were analyzed to ensure that densitometry was within the linear range. To assess Gag processing (%p30 processing) in cells, the percentage of cellular p30 divided by Pr65 and p30 in cells was calculated. 

### 2.6. Statistical Analysis

Welch’s *t*-test was used to compare the scores in the two groups to assess virus release efficiency and Gag processing in the cells. Student’s *t*-test was used to compare the colocalization signals in the cells.

## 3. Results

**Figure 1:**
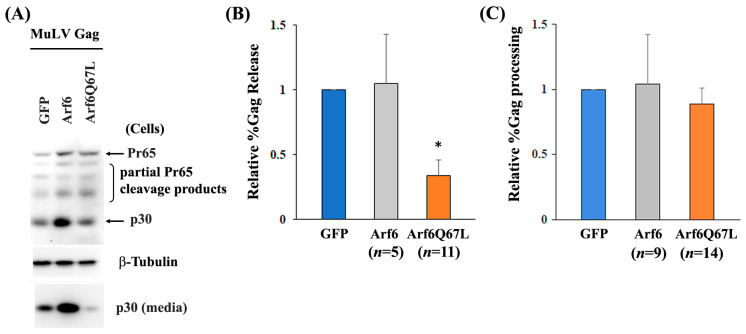
Arf6Q67L impaired release of MuLV Gag. The MuLV Gag/Pol expression vector was co-transfected with the plasmids expressing GFP, Arf6-CFP (Arf6), or Arf6Q67L-CFP (Arf6Q67L) into 293T cells. (**A**) The cells and media were harvested 48 h after transfection. MuLV Gag proteins in the cells and viruses were analyzed by western blots with the serum against p30. β-Tubulin was detected as a loading control. (**B**) The relative viral release efficiencies (%Gag release) were quantified with an immuno-densitometry software, and the means ± SD are shown (*n*, the number of independent experiments). (**C**) The relative %Gag processing was calculated, and the means ± SD are shown (*n*, the number of independent experiments). * Comparison of the viral release efficiencies between the groups expressing GFP and Arf6Q67L, *p* < 5 × 10^−6^ (Welch’s *t*-test).

To investigate the role of Arf6 in the release of MuLV Gag, 293T cells were co-transfected with a MuLV Gag/Pol expression vector and the plasmids expressing Arf6. Western blots detected Gag proteins released from the transfected cells using the rabbit serum against MuLV Capsid/p30. Induction of Arf6Q67L did not show substantial differences in the expression of cellular Pr65 and p30 but impaired release of MuLV p30 into the media (Figure 1). The expression of Arf6 increased intracellular Gag (Pr65, partial Pr65 cleavage products, and p30) and Gag in media and did not show major changes in viral release efficiency (%Gag release) (Figure 1). A comparison of the percentages of cellular p30 divided by the sum of Pr65 and p30 showed that both Arf6 and Arf6Q67L did not show significant effects on Gag processing in the cells (Figure 1). The experiments using a plasmid encoding HIV-1 Gag/Pol and Arf6Q67L showed similar results (Figure S1), which were consistent with the report of using the HIV-1 molecular clone pNL4-3 [19].

**Figure 2:**
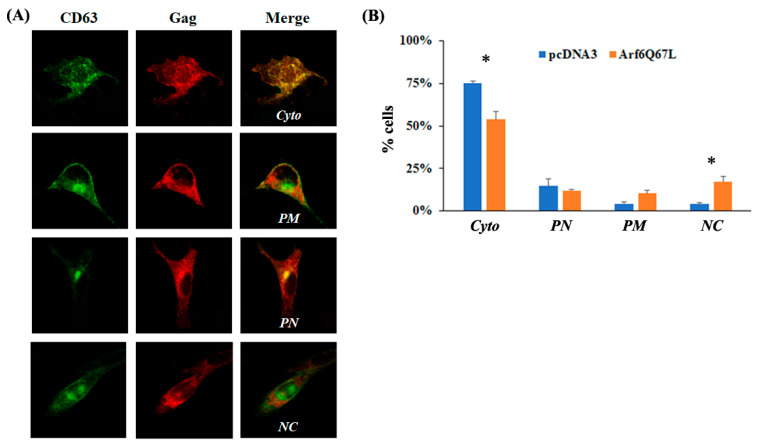
Arf6Q67L decreased the colocalization of CD63 and MuLV Gag. The plasmids expressing CD63 conjugated with GFP (green), Arf6Q67L-HA, and MuLV Gag/Pol were transfected into NIH3T3 cells. The cells were fixed at 48 h post-transfection. The Gag was stained with the serum against p30 and anti-rabbit IgG conjugated with Alexa 555 (red). Localization of the proteins was analyzed by the confocal microscope Zeiss LSM800. (**A**) The cells showing colocalization of CD63 and MuLV Gag in the cytoplasm, on the plasma membranes, and at perinuclear regions, as well as no colocalization were classified as Cyto, *PM*, *PN,* and *NC*. (**B**) The percentage of the cells showing each phenotype is indicated (means ± SD of two independent experiments). * *p* < 0.05 (Student’s *t*-test).

**Figure 3:**
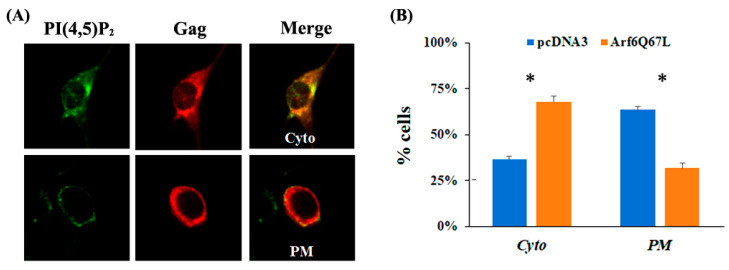
Arf6Q67L accumulated PI(4,5)P2 and MuLV Gag in the cytoplasm. The plasmids expressing the PH domain of phospholipase C1 conjugated with GFP (green), Arf6Q67-HA, and MuLV Gag/Pol were transfected into NIH3T3 cells. At 48 h post-transfection, the cells were fixed, and Gag was stained with the serum against p30 and anti-rabbit IgG conjugated with Alexa 555 (red). Localization of the proteins was analyzed by the confocal microscope Zeiss LSM800. (**A**) The cells showing colocalization of phospholipase C1 and MuLV Gag in the cytoplasm and on the plasma membranes were classified as *Cyto* and *PM*. (**B**) The percentage of the cells showing each phenotype is indicated (means ± SD of two independent experiments). * *p* < 0.05 (Student’s *t*-test).

The previous studies using Arf6Q67L decreased virus particle production through the accumulation of HIV-1 and HIV-2 Gag in the PI(4,5)P_2_-enriched intracellular vesicles [19,20]. MuLV Gag localizes in late endosomal membranes, multivesicular bodies, and the plasma membranes [21,22], and eventually produces viral particles. To understand how Arf6Q67L restricted MuLV Gag release and colocalization of Gag and CD63, a marker for late endosomes/multivesicular bodies [23], or PI(4,5)P_2_, was determined by confocal microscopy. The cells were transfected with the plasmids expressing CD63 conjugated with GFP and MuLV Gag. The cells were classified into four phenotypes according to the localization of the molecules; colocalization signals were (1) dispersed mainly in the cytoplasm (*Cyto*), (2) at both the plasma membrane and cytoplasm (*PM*), (3) intensive at the perinuclear area (*PN*), and (4) not observed (no colocalization, *NC*) (Figure 2A). In the cells transfected with the control plasmids, MuLV Gag (red) and CD63 (green) predominantly colocalized in the cytoplasm, and a small portion of cells showed other phenotypes. Arf6Q67L decreased the number of cells showing colocalization of Gag and CD63 in the cytoplasm and increased the number of cells showing no colocalization of the molecules (Figure 2B). To monitor PI(4,5)P_2_, the plasmid expressing PH domain of phospholipase C1 (PLC1) conjugated with GFP that binds to PI(4,5)P_2_ with high affinity was expressed in the cells. PI(4,5)P_2_ (green) was mainly observed at the plasma membrane in the control cells but was redistributed to the cytoplasm in the cells expressing Arf6Q67L. PI(4,5)P_2_ also colocalized with Gag (red), and the colocalization of PI(4,5)P_2_ and Gag shifted from the plasma membrane to the cytoplasm (Figure 3A,B). 

**Figure 4:**
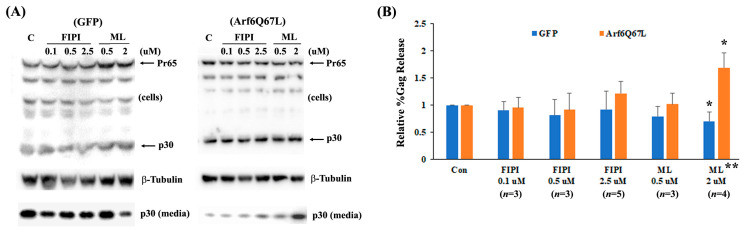
ML299 affected MuLV Gag release with and without Arf6Q67L. The MuLV Gag/Pol expression vector was co-transfected with the plasmids expressing GFP or Arf6Q67L-CFP into 293T cells. (**A**) The media were replaced at 24 h post-transfection, and the cells were treated with the PIP5K inhibitors, FIPI, and ML299 (ML). Both the cells and media were harvested after 24 h of further incubation. MuLV Gag proteins in the cells and viruses were analyzed by western blots with the serum against p30. β-Tubulin was detected as a loading control. (**B**) The relative viral release efficiencies (%Gag release) were quantified, and the means ± SD are shown (*n*, the number of independent experiments). * Comparison of the groups between the cells with and without chemical treatment, *p* < 0.05 (Welch’s *t*-test). ** Comparison of the groups between the cells expressing GFP and Arf6Q67L, *p* < 0.05 (Welch’s *t*-test).

**Figure 5:**
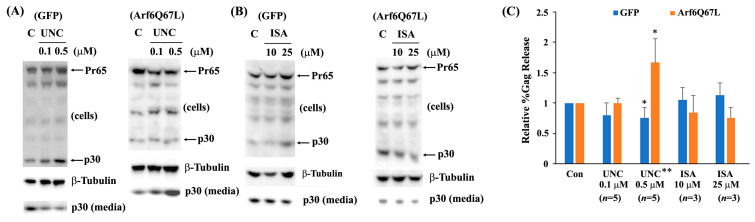
UNC3230 affected MuLV Gag release with and without Arf6Q67L. The MuLV Gag/Pol expression vector was co-transfected with the plasmids expressing GFP or Arf6Q67L-CFP into 293T cells. At 24 h post-transfection, the media were replaced, and the cells were treated with the (**A**) UNC3230 (UNC) and (**B**) ISA-2011B, (ISA). Both the cells and media were harvested after 24 h of further incubation. MuLV Gag proteins in the cells and viruses were analyzed by western blots with the serum against p30. β-Tubulin was detected as a loading control. (**C**) The relative viral release efficiencies (%Gag release) were quantified, and the means ± SD are shown (*n*, the number of independent experiments). * Comparison of the groups between the cells with and without chemical treatment, *p* < 0.05 (Welch’s *t*-test). ** Comparison of the groups between the cells expressing GFP and Arf6Q67L, *p* < 0.05 (Welch’s *t*-test).

Since Arf6 produces PI(4,5)P_2_ through PLD1 and PIP5K, Gag release was assessed using the chemical inhibitors, FIPI, ML299, UNC3230, and ISA-2011B. FIPI and ML299 are potent and selective inhibitors of mammalian PLD1 and PLD2 [24,25]. Cells were transfected with a MuLV Gag/Pol expression vector and either a plasmid encoding GFP (control) or Arf6Q67L, and then treated with the chemical inhibitors. FIPI (0.1–2.5 µM) showed a minor reduction (without statistical significance) in virus release efficiency from the cells expressing GFP and a modest increase in virus release from the cells expressing Arf6Q67L (Figure 4A,B). ML299 recovered virus release from the cells expressing Arf6Q67L and impaired virus release from the control cells. ISA-2011B is an inhibitor of PIP5Kα, and UNC3230 is an inhibitor of PIP5Kγ with no inhibition of PIP5Kα at concentrations up to 10 μM [26]. UNC3230 increased virus release from the cells expressing Arf6Q67L and decreased virus release in the control cells (Figure 5A,C). In contrast, ISA-2011 did not show significant effects on MuLV release from the cells regardless of Arf6Q67L expression (Figure 5B,C). Treatment with these chemicals showed modest effects on Gag processing and on the viability of the 293T cells based on the β-Tubulin signals in Figure 4 and Figure 5. The data imply that both PLD and PIP5K could affect MuLV release. 

**Figure 6:**
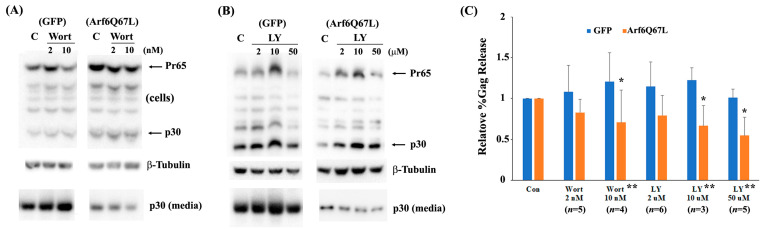
PI3K inhibitors reduced MuLV Gag release from the cells expressing Arf6Q67L. The MuLV Gag/Pol expression vector was co-transfected with the plasmids expressing GFP or Arf6Q67L-CFP into 293T cells. The media were replaced at 24 h post-transfection, and the cells were treated with (**A**) wortmannin (Wort) and (**B**) LY294002 (LY). Both the cells and media were harvested after 24 h of further incubation. MuLV Gag proteins in the cells and viruses were analyzed by western blots with the serum against p30. β-Tubulin was detected as a loading control. C, control (DMSO). (**C**) The relative viral release efficiencies (%Gag release) were quantified, and the means ± SD are shown (*n*, the number of independent experiments). * Comparison of the groups between the cells with and without chemical treatment, *p* < 0.05 (Welch’s *t*-test). ** Comparison of the groups between the cells expressing GFP and Arf6Q67L, *p* < 0.05 (Welch’s *t*-test).

**Figure 7:**
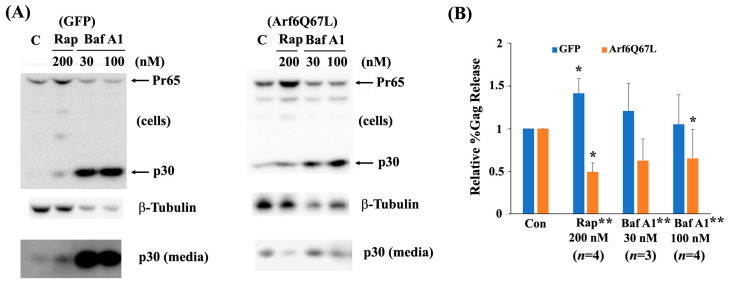
Rapamycin and bafilomycin A1 reduced MuLV Gag release from the cells expressing Arf6Q67L. (**A**) The MuLV Gag/Pol expression vector was co-transfected with the plasmids expressing GFP or Arf6Q67L-CFP into 293T cells. The media were replaced at 24 h post-transfection, and the cells were treated with rapamycin (Rap) and bafilomycin A1 (BafA1). Both the cells and media were harvested after 24 h of further incubation. MuLV Gag proteins in the cells and viruses were analyzed by western blots with the serum against p30. β-Tubulin was detected as a loading control. (**B**) The relative viral release efficiencies (%Gag release) were quantified, and the means ± SD are shown (*n*, the number of independent experiments). * Comparison of the groups between the cells with and without chemical treatment, *p* < 0.05 (Welch’s *t*-test). ** Comparison of the groups between the cells expressing GFP and Arf6Q67L, *p* < 0.05 (Welch’s *t*-test).

Autophagy is an evolutionarily conserved degradative system that is an essential cellular process for breaking down defective organelles and aggregated proteins via the lysosomal pathways. This process involves various steps, such as the formation of phagophores, autophagosomes, and autolysosomes [27]. Autophagy is also considered a part of innate immunity since it can degrade viral components and host factors during the early and late phases of infection [28]. Arf6 controls autophagy by producing PI(4,5)P_2_ and its downstream mediator phosphatidylinositol (3,4,5)-trisphosphate [3]. Since Arf6Q67L impaired Gag release (Figure 1), we hypothesized that Arf6Q67L trapped Gag in unique cellular components and reduced virus release efficiency by Gag degradation via autophagy or other systems. To assess the hypothesis, cells were treated with the chemicals widely used to modulate autophagy activity. PI3K inhibitors LY294002 and wortmannin and bafilomycin A1 impair autophagy activity by preventing autophagosome formation and fusion of autophagosome with lysosome [29]. Rapamycin, on the other hand, abrogates the mTOR activation and induces autophagy. LY294002 decreased virus release efficiency in the cells expressing Arf6Q67L, but the chemical did not affect Gag release in the control cells (Figure 6A,C). Comparison of the relative %Gag release between the cells expressing GFP and Arf6Q67L was also significant. Similar to LY294002, wortmannin exhibited a modest increase in MuLV Gag release (relative %Gag release to the cells treated with DMSO) in the control cells and a decrease in MuLV Gag release from the cells expressing Arf6Q67L at 10 nM (Figure 6B,C). LY294002 has a tendency to increase %Gag processing in both the cells expressing GFP and Arf6Q67L, but the effects were not observed in the cells treated with wortmannin. Rapamycin increased intracellular Gag regardless of Arf6Q67L expression and did not affect Gag processing in the cells (Figure 7A). While rapamycin increased virus release efficiency in the control cells, %Gag release was decreased in the cells expressing Arf6Q67L (Figure 7A,B). Treatment with bafilomycin A1 (30 nM and 100 nM) resulted in a significant increase in intracellular p30 and released p30 from the control cells, without affecting viral release efficiency (Figure 7A,B). This suggested that V-ATPase may affect viral maturation or protease activities. In contrast, bafilomycin A1 (30 nM and 100 nM) increased intracellular p30 and reduced p30 released into media from the cells expressing Arf6Q67L (Figure 7A,B). Similar trends were observed in the cells treated with lower doses (3 nM and 10 nM) of bafilomycin A1, but only the control cells showed an increase in intracellular p30 under the conditions (Figure S2). 

A previous report demonstrated that Arf6Q67L blocked the formation of early autophagic structures in HeLa cells [12]. Therefore, we monitored LC3 levels in the 293T cells transfected with Arf6Q67L. The expression of LC3-II/β-Tubulin was comparable between the cells expressing GFP and Arf6Q67L-CFP (Figure S3A). The relative expression of LC3-II and p62 normalized with β-Tubulin showed similar trends between the cells expressing GFP and Arf6Q67L under treatment of the autophagy modulators (Figure S3B). The data implied that the differences in viral release efficiency between the cells expressing GFP and Arf6Q67L can be attributed to the alteration of the Gag release pathway by Arf6Q67L rather than different autophagic flux in the cells treated with the chemical inhibitors.

**Figure 8:**
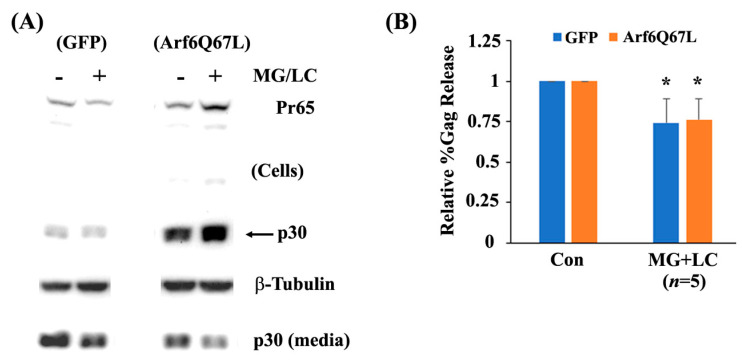
Proteasome inhibitors reduced MuLV Gag release. (**A**) The MuLV Gag/Pol expression vector was co-transfected with the plasmids expressing GFP or Arf6Q67L-CFP into 293T cells. The media were replaced at 48 h post-transfection, and the cells were treated with MG132 (MG) and lactacystin (LC). Both the cells and media were harvested after 8 h of further incubation. MuLV Gag proteins in the cells and viruses were analyzed by western blots with the serum against p30. β-Tubulin was detected as a loading control. (**B**) The relative viral release efficiencies (%Gag release) were quantified, and the means ± SD are shown (*n*, the number of independent experiments). Comparison of the groups between the cells with and without chemical treatment, * *p* < 0.05 (Welch’s *t*-test).

The proteasome is the protein complex that breaks down proteins through ubiquitination, followed by proteolysis [30]. Along with autophagy, the proteasome is essential for maintaining cellular activities and is also associated with the replication of many viruses [31]. We tested whether the proteasome is involved in the release of MuLV Gag in the cells expressing Arf6Q67L using the proteasome inhibitors lactacystin and MG132 [30]. Treatment with individual proteasome inhibitors at concentrations of 0.1–10 µM for 24 h did not have a major effect on the production of Pr65, cleavage of p30, or virus release in cells expressing GFP and Arf6Q67L. However, co-treatment with the inhibitors impaired virus release from the cells expressing GFP (*p* < 0.01) and Arf6Q67L (*p* < 0.05) (Figure 8), suggesting the involvement of the proteasomes in both Arf6-dependent and Arf6-independent viral release pathways.

## 4. Discussion

Overexpression of Arf6 increased the levels of intracellular Gag but did not affect MuLV release, in contrast to the cells expressing Arf6Q67L that severely reduced MuLV Gag release (Figure 1). The data suggest that enhancement of PI(4,5)P_2_ itself would not affect Gag release from the cells, and the restriction of MuLV release by Arf6Q67L could mainly be attributed to the redistribution of the molecules required for Gag trafficking in the cells. We found that the expression of Arf6Q67L changed the localization of PI(4,5)P_2_ from the plasma membrane to the cytoplasm, and this shift was accompanied by less colocalization between Gag and CD63 (Figure 2) and redistribution of MuLV Gag to the intracellular regions with accumulated PI(4,5)P_2_ (Figure 3). The observations are consistent with the trafficking of HIV-1 Gag in HeLa cells expressing Arf6Q67L that showed redistribution and partial colocalization of GFP-tagged PH domain from phospholipase Cδ1P (a marker for PI(4,5)P_2_) in the intracellular vesicular structures [19]. Overexpression of 5ptaseIV inhibited HIV and MuLV Gag release. However, the restriction of viral release by 5ptaseIV was modest when the HIV had a deletion in the matrix domain and the MuLV had mutations in the polybasic region [19,32]. These data suggest that both the direct interaction between PI(4,5)P_2_ and Gag and the localization of PI(4,5)P_2_ could determine Gag trafficking in the cells and the subsequent viral release from the plasma membrane. 

Arf6 activates both PLD and PIP5K and facilitates the production of PI(4,5)P_2_ [3,6]. Our experiments with chemical inhibitors for PLD1/2 and PIP5Ks showed that ML299 and UNC3230 partially recovered viral release from the cells expressing Arf6Q67L (Figure 4 and Figure 5). Restoration of viral production could be explained by fewer Gag molecules trapped in the PI(4,5)P_2_-enriched compartments in the cytoplasm through attenuation of PI(4,5)P_2_ overproduction in the cells expressing Arf6Q67L. Both ML299 and UNC3230 also showed a reduction of Gag release in the control cells (Figure 4 and Figure 5). The data imply that maintenance of certain levels of PI(4,5)P_2_ and activities of PLDs and PIP5Kγ could be required for efficient MuLV Gag release from the cells in their native condition (cells expressing GFP). The IC_50_ values of ML299 are 6 nM (PLD1) and 12 nM (PLD2), and the IC_50_ values of FIPI are 25 nM (PLD1) and 20 nM (PLD2), respectively [24,25]. UNC3230 is an inhibitor for PIP5Kγ with no inhibition of PIP5Kα at concentrations up to 10 μM [26]. Our data suggested that PLD1 and PIP5Kγ would be the key drivers that maintain MuLV Gag release from 293T cells in Arf6-dependent and/or Arf6-independent manners. In pheochromocytoma cells, PC12, overexpression of PLD1—but not PLD2—stimulated a PI(4,5)P_2_-dependent exocytosis without reorganization of the actin cytoskeleton [33]. PLD1 and PLD2 are involved in different steps of exocytosis by each regulating movement of secretory granules/vehicle to the periphery (PLD1) and fusion of granules with the plasma membrane (both PLD1 and PLD2) in mast (RBL-2H3), pancreatic β (MIN6) and pituitary cells [34,35,36]. PLD1-dependent exocytosis pathway could be overlapped with that for MuLV release, and it would be regulated by Arf6.

**Figure 9:**
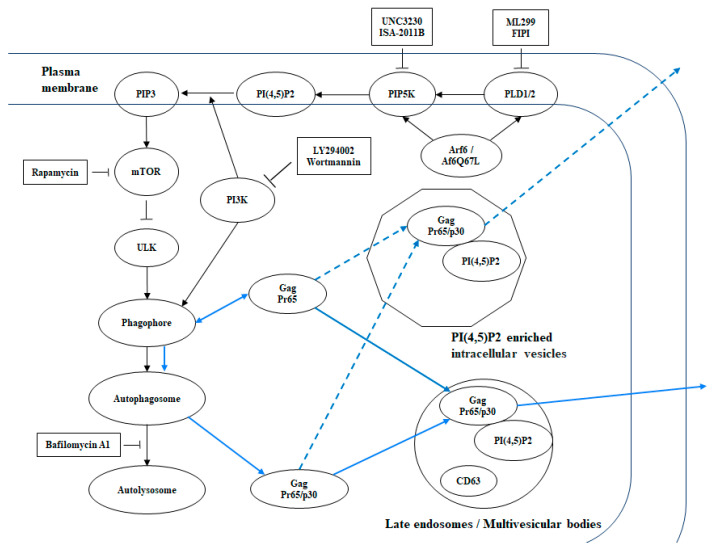
Arf6 signaling pathway, the inhibitors used in this study, and a model of MuLV Gag release. Some effector molecules in the Arf6 signaling pathway are shown with the inhibitors used in the study. The blue arrows depict the trafficking of MuLV Gag in the model. The solid and broken arrows show the virus release pathways under the controlled (solid) and overactivated (broken) Arf6 conditions. PIP3, phosphatidylinositol (3,4,5)-trisphosphate, ULK, Unc51-like kinase.

Our pharmacological approach provided insights into the role of the proteins relevant to autophagy regulation in MuLV release. The data also contrasted viral release pathways in native conditions (cells transfected with a GFP expression vector) and with expression of Arf6Q67L. We initially hypothesized that the inhibition of PI3K and V-ATPase would recover Gag release from the cells expressing Arf6Q67L by preventing Gag degradation through autophagy. Contrary to our hypothesis, the approach to inhibit PI3K, mTOR, and V-ATPase showed further impairment of Gag release from the cells expressing Arf6Q67L, while mTOR inhibition increased Gag release in the control cells (Figure 6 and Figure 7). The current data using wortmannin and LY294002 suggest that PI3K activities such as endosomal sorting and recycling and formation of phagocytosis [37] did not have major impacts on the efficiency of MuLV Gag release in native conditions. Bafilomycin A1, which inhibits autophagosome-lysosome fusion and lysosome acidification regulated by V-ATPase [38], showed an increase in intracellular p30, LC3-II, p62, and the level of p30 released into media without changing %Gag release in the control cells (Figure 7 and Figure S3). The data imply that autophagosome-lysosome fusion impaired total p30 production by Gag degradation but did not interfere with Gag cleavage and release of p30 from the plasma membrane or other membrane compartments. Since MuLV release was reduced by the inhibitors for PI3K, mTOR, and V-ATPase in the cells expressing ArfQ67L, the lack of the activities regulated by these molecules could induce redistribution of Gag to the PI(4,5)P_2_-enriched compartments in the cytoplasm and could interfere with the formation and trafficking of vehicles to export Gag from the PI(4,5)P_2_-enriched compartments (Figure 9). 

Rapamycin has received considerable attention in various fields, such as cancer, antiaging, and infection, due to the multifunction of mTOR in cells. Regarding the retroviral life cycle, chronic treatment of the cells with rapamycin interfered with HIV-1 entry by downregulating CCR5 and with HIV-1 basal transcription from the LTR [39,40], which was corroborated with the research showing that HIV enhanced the mTOR activity transiently to upregulate Gag synthesis [41]. Rapamycin also suppressed expression from the HTLV-1 LTR through NF-kB [42]. In contrast to its inhibitory effects on viral replication, Kyei et al. reported that rapamycin enhanced HIV-1 release from macrophages without affecting the release of LDH [43]. This effect was not observed with HIV-1 lacking Nef in macrophages and wild-type HIV-1 in HeLa or H8T cell lines, suggesting that both cellular and viral factors could affect the effects of rapamycin. We found here that rapamycin increased intracellular Gag (both Pr65 and p30) and virus release efficiency in native conditions (Figure 7). The data imply that mTOR suppressed Gag production/stability and virus release in MuLV. Since the activity of mTORC1 is negatively regulated by AMP-activated protein kinase, tuberous sclerosis protein complex, and GATOR-Rag GTPase, which are induced by a lack of growth factors and energetic stresses [44], MuLV might have advantages in maintaining viral production during adverse nutritional conditions. 

We investigated if Arf6Q67L affects viral release pathways regulated by the proteasome using the proteasome inhibitors, MG132 and lactacystin. We found here that co-treatment of the proteasome inhibitors reduced MuLV Gag release from the cells regardless of Arf6Q67L expression (Figure 8). Our data were consistent with the previous reports showing impairment of HIV, MuLV, and Rous sarcoma virus release after treatment with several proteasome inhibitors [15,45,46]. Depletion of free ubiquitin by the proteasome inhibitors prevented ubiquitination on retroviral Gag and could impair the vacuolar protein sorting pathway, resulting in arresting the viruses at a budding step [47,48]. Our data imply that redistribution of PI(4,5)P_2_ and signals induced by Arf6Q67L would not have major impacts on ESCRT-dependent MuLV release that could be interfered with by the proteasome inhibitors. The processing of MuLV Gag (ratio of intracellular p30 to Pr65) in the treated cells was not affected by the proteasome inhibitors (Figure 8), which was in contrast to the reduction of HIV Gag processing (ratio of intracellular p24 to Pr55) in the cells treated with 10 µM of each MG132 and lactacystin [15]. Since HIV-1 protease activity was not directly affected by the proteasome inhibitors in vitro, Gag processing might be regulated through indirect attenuation of protease activity by the proteasome inhibitors or another mechanism in cells [15]. The difference in Gag processing between the viruses implies that the molecules required for HIV maturation could be more sensitive to proteasome inhibitors than those of MuLV, and this idea could be supported in part by the differences between HIV and MuLV proteases in their distantly related specificities [49]. Since inhibition of PLD, PIP5K, PI3K, and mTOR did not change Gag processing significantly (Figure 4, Figure 5, Figure 6 and Figure 7), these molecules are not likely to control the activity of MuLV protease.

While mild pharmacological treatments were selected to avoid nonspecific effects, our current system might have some limitations due to the potential multi-inhibitory effects of the chemical inhibitors. Nevertheless, this study using a constitutively active form of Arf6 has disclosed the involvement of Arf6, PLD, PIP5K, and PI(4,5)P_2_ on MuLV Gag release and has outlined viral release pathways sensitive to the chemicals modulating PI3K, mTOR, V-ATPase, and proteasome in native condition and with expression of Arf6Q67L. The significance of these molecules in the early phase of viral infection and translation has been extensively investigated [50,51,52], but their effects on viral release remain largely unknown. Our data provided new insights into retroviral life cycles. We believe the system could be useful for investigating how Arf6 and its signaling molecules regulate virus release in combination with inhibitors, RNA interference, and gene modification techniques.

## Data Availability

The data presented in this study are available from the corresponding author upon request.

## Supplementary Materials

The following supporting information can be downloaded at: https://www.mdpi.com/article/10.3390/cells13030270/s1, Figure S1: Arf6Q67L impaired release of HIV Gag; Figure S2: Effects of low doses of bafilomycin A1 on intracellular Gag and Gag release; Figure S3: Effects of Arf6Q67L on autophagy flux affected by the inhibitors.
