# Supplementary material for: Evidence for Involvement of ADP-Ribosylation Factor 6 in Intracellular Trafficking and Release of Murine Leukemia Virus Gag
Source: Cells. 2024 Jan 31;13(3):270. doi: 10.3390/cells13030270 (PMC10854678; doi:10.3390/cells13030270)
Supplement: Supplementary file 1 [file cells-13-00270-s001.zip › cells-2788704-supplementary.pdf]

Article

Evidence for Involvement of ADP-Ribosylation Factor 6 in Intracellular Trafficking and Release of Murine Leukemia Virus Gag

Hyokyun Kang [1], Taekwon Kang [1], Lauryn Jackson [1], Amaiya Murphy [1] and Takayuki Nitta [1,2,*]

[1] Department of Biology, Savannah State University, Savannah, GA 31404, USA; hkang36906@med.lecom.edu (H.K.); tkang@student.savannahstate.edu (T.K.); ljacks63@student.savannahstate.edu (L.J.); amurph16@student.savannahstate.edu (A.M.)

[2] Department of Molecular Biology and Biochemistry, Cancer Research Institute, University of California, Irvine, CA 92697, USA

* Correspondence: nittat@savannahstate.edu; Tel.: +1-912-358-4459

Supplemental Materials.

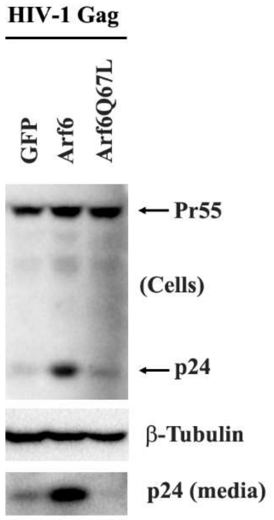

Figure S1. Arf6Q67L impaired release of HIV Gag. The HIV-1 Gag/Pol expression vector was co-transfected with the plasmids expressing GFP, Arf6-CFP or Arf6Q67L-CFP into 293T cells. The cells and media were harvested 48 hours after transfection and the samples were analyzed by western blots with anti-HIV p24 and anti-β-Tubulin antibodies.

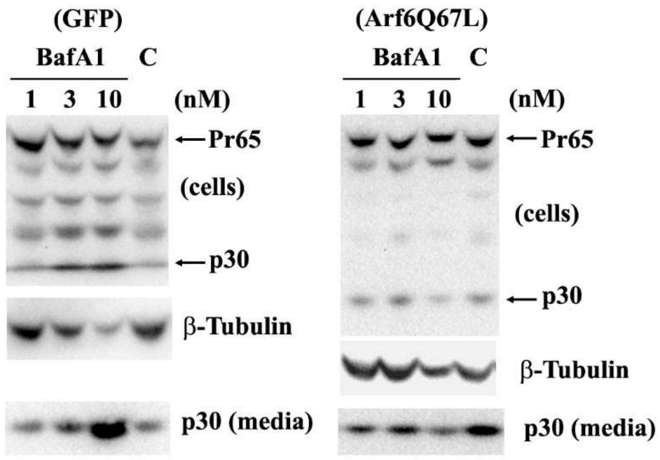

Figure S2. Effects of low doses of bafilomycin A1 on intracellular Gag and Gag release. The MuLV Gag/Pol expression vector was co-transfected with the plasmids expressing GFP or Arf6Q67L-CFP into 293T cells. The media were replaced at 24 hours post-transfection, and the cells were treated with 1, 3 and 10 nM of bafilomycin A1. The cells and media were harvested after 24 hours of further incubation. MuLV Gag proteins in the cells and viruses were analyzed by western blots with the serum against p30. β-Tubulin was detected as a loading control.

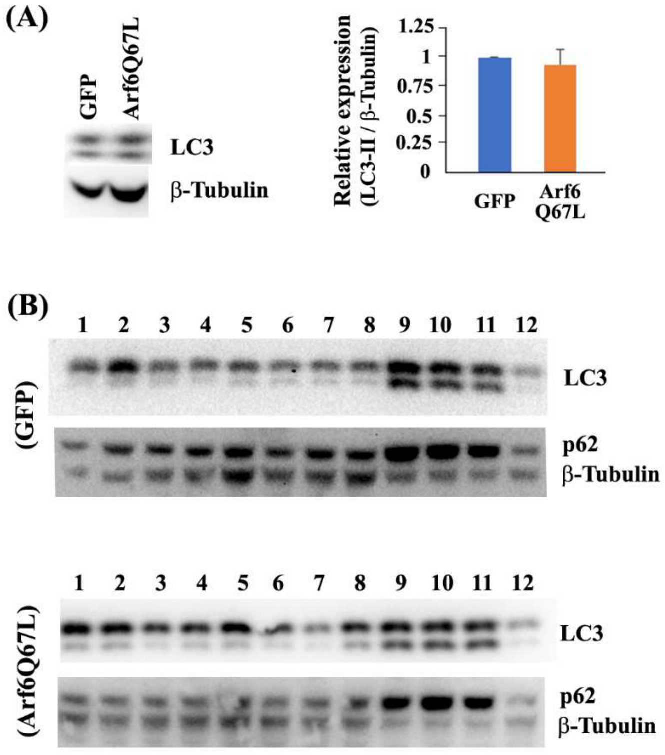

Figure S3. Effects of Arf6Q67L on autophagy flux affected by the inhibitors. The plasmids expressing GFP or Arf6Q67L-CFP were transfected into 293T cells. (A) LC3 and β-Tubulin were detected in the cells 48 hours post-transfection. The relative expression of LC3-II normalized by β-Tubulin was quantified with an immuno-densitometry software (means ± SD of 4 independent experiments). (B) The doses of the chemical inhibitors were given to the cells 24 hours post-transfection of GFP and Arf6Q67L-CFP. The cells were collected at 48 hours post-transfection and expressions of LC3, p62/SQSTM1 and β-Tubulin were detected by western blots. 1, DMSO, 2-3, wortmannin (2, 10 μM), 4-6, LY290042 (2, 10, 50 μM), 7-11, bafilomycin A1 (1, 3, 10, 30, 100 nM), 12, rapamycin (200 nM).
